# Review of light parameters and photobiomodulation efficacy: dive into complexity

**DOI:** 10.1117/1.JBO.23.12.120901

**Published:** 2018-12-11

**Authors:** Randa Zein, Wayne Selting, Michael R. Hamblin

**Affiliations:** a University of Genoa, Department of Surgical Science and Integrated Diagnostics, Genoa, Italy; b Massachusetts General Hospital, Wellman Center for Photomedicine, Boston, Massachusetts, United States; c Harvard Medical School, Department of Dermatology, Boston, Massachusetts, United States; d Harvard-MIT Division of Health Sciences and Technology, Cambridge, Massachusetts, United States

**Keywords:** photobiomodulation, low-level laser therapy, parameters, mitochondrial numbers, effective and ineffective studies

## Abstract

Photobiomodulation (PBM) therapy, previously known as low-level laser therapy, was discovered more than 50 years ago, yet there is still no agreement on the parameters and protocols for its clinical application. Some groups have recommended the use of a power density less than $100\;{\mathrm{mW}/\mathrm{cm}}^{2}$ and an energy density of 4 to $10\;{J/\mathrm{cm}}^{2}$ at the level of the target tissue. Others recommend as much as $50\;{J/\mathrm{cm}}^{2}$ at the tissue surface. The wide range of parameters that can be applied (wavelength, energy, fluence, power, irradiance, pulse mode, treatment duration, and repetition) in some cases has led to contradictory results. In our review, we attempt to evaluate the range of effective and ineffective parameters in PBM. Studies *in vitro* with cultured cells or *in vivo* with different tissues were divided into those with higher numbers of mitochondria (muscle, brain, heart, nerve) or lower numbers of mitochondria (skin, tendon, cartilage). Graphs were plotted of energy density against power density. Although the results showed a high degree of variability, cells/tissues with high numbers of mitochondria tended to respond to lower doses of light than those with lower number of mitochondria. Ineffective studies in cells with high mitochondrial activity appeared to be more often due to over-dosing than to under-dosing.

## 1 Introduction

Since Mester,^1^^,^^2^ in 1968, accidently discovered the positive effect of a ruby laser beam on hair growth and wound healing in mice, researchers have attempted to uncover the scientific basis for this phenomenon as well to establish the range of optical exposure parameters that lead to successful clinical outcomes. The possibility of stimulating a wide range of cells to improve wound healing and cellular growth has created a science referred to as low-level laser therapy (LLLT) or photobiomodulation therapy (PBMT). As an understanding of basic concepts has emerged, the very wide range of factors contributing to positive outcomes in some cases and negative outcomes in others has stymied the development of definitive protocols.

The multitude of variables to be considered is formidable. More than 1000 research articles have reported that a range of factors can apparently affect the chances of success including wavelength, energy density, power density, total energy, total power, pulse structure, spot size, tissue absorption characteristics, and treatment repetition regimen. Further parameters of lesser importance requiring both control and study are use of combination wavelengths, delivery method (contact, punctual, broad beam), duration of treatment, inadvertent heating of tissue and even whether the source of photons is a laser, light-emitting diode (LED), or broad-spectrum light from a lamp.^3^^,^^4^

It has become apparent that, in order to achieve positive results with PBM, each of these dosimetric parameters must be controlled within a limited range of values. Of the many studies that have been conducted over the past 50 years, a number have attempted to determine the relative contribution of individual parameters to successful outcomes.

Consensus has (almost) been reached on one of the most important concepts in PBM. The so-called Arndt–Schultz law was originally proposed near the end of the 19th century. It states in original form that “For every substance, small doses stimulate, moderate doses inhibit, and large doses kill.”^5^ This concept^6^ also forms the basis of the science of “hormesis,” as reviewed by Calabrese and Mattson^7^

Pharmacological agents used at a therapeutic dose can be very beneficial while the same drug administered at a higher dose may be catastrophic. For many years, this Arndt–Schultz law has been used as a convenient concept to explain the cellular and tissue interactions with light.

Briefly, this law, when applied to PBM, states that, at very low levels of irradiation, photons are absorbed by subcellular chromophores present inside intracellular organelles, most notably, mitochondria. Absorption of energy by cytochrome C oxidase (CCO) in the mitochondrial respiratory chain is the primary initiating interaction triggering PBM effects.^8^ Both adenosine triphosphate (ATP) production and oxygen consumption by the cells increase. This may lead to changes in nitric oxide (NO) levels, activation of secondary messenger pathways, activation of transcription factors, and growth factor production.^9^ At this very low level, energy is absorbed by the cell but at such low amounts of energy that there are no observable gross changes (temperature or photochemical damage).

As the number of absorbed photons increases, stimulation of cellular metabolism, as noted above, begins to affect cellular activity, producing positive PBM effects. Both the number of photons and rate at which they are delivered has a significant influence on the response.^10^^,^^11^

As the number of photons increases beyond a particular level, the cellular stimulation disappears, and if the number of photons is even further increased, inhibition and cellular damage occurs. Current theories suggest that the mitochondrial membrane potential having reached a maximum at the optimum dose declines back to baseline and can be lowered below baseline by excessive doses of light.^12^ ATP reserves within the cell begin to be depleted by excessive doses of light compromising the positive cellular function. Production of excessive reactive oxygen species (ROS), which can be toxic, release of excessive free NO, which can damage cells, and activation of a cytotoxic mitochondrial-signaling pathway leading to apoptosis are also possible theories. At still higher levels of irradiation, depletion of cellular energy reserves or excess levels of the factors noted above become so significant that cellular metabolism falls below normal intrinsic levels and function is actually inhibited eventually leading to cell death.

This concept, represented by the Arndt–Schultz law of biphasic dose response, has become the foundational concept of PBM. However, the appropriate range of values of fluence and irradiance at which these significant transitions occur are not widely agreed upon. Numerous studies suggest that fluences ranging from 3 to $10\;{J/\mathrm{cm}}^{2}$, at the cellular level, will produce the desired stimulation of metabolic activity.^13^^,^^14^

While this protocol has become widely accepted, some studies suggest that biostimulation will occur in the range of 0.5 to $1\;{J/\mathrm{cm}}^{2}$ on an open wound and in the range of 2 to $4\;{J/\mathrm{cm}}^{2}$ to a target through overlaying skin.^15^ Another respected source suggests that doses used for superficial targets tend to be in the region of $4\;{J/\mathrm{cm}}^{2}$ with a range of 1 to $10\;{J/\mathrm{cm}}^{2}$.^16^^–^^18^ Doses for deeper-seated targets should be in the 10 to $50{/\mathrm{cm}}^{2}$ range.^19^^–^^21^

While many studies have shown a positive effect of PBM,^17^^,^^18^^,^^21^ a number have failed to show a benefit^22^^,^^23^ and, in fact, some reports have shown negative outcomes at what are reported to be the same parameters of irradiation as other positive studies. Unfortunately, in many of the historical studies, important laser parameters were omitted or incorrectly presented.

Often, laser output total power is reported without consideration of the spot diameter at the surface of the target tissue. Therefore, power density, the most relevant parameter, is not reported and results are, predictably, inconsistent.

Sometimes the distribution of energy across the tissue surface is not noted in published studies introducing profound errors. As an example, most lasers are designed to emit in the ${\mathrm{TEM}}_{00}$ mode, which produces a Gaussian distribution of beam profile. By mathematical definition, cells in the exact center of the beam will be irradiated at precisely twice the indicated average output power while cells at the periphery of the irradiation spot will only receive about 13% of that power. If irradiation were to be delivered for 30 s, cells at the beam center would receive an energy dose of $6\;{J/\mathrm{cm}}^{2}$ while those at the periphery would receive $0.39\;{J/\mathrm{cm}}^{2}$. Obviously, the cellular response, taking into account the Arndt–Schultz law, will be different in each of these tissues. This could result in a conclusion of no-effect, positive effect, or negative effect, depending on which cells were observed in the analysis phase of the study.

Another basic concept that has been suggested to be relevant to the successful application of PBM is the Roscoe–Bunsen law of reciprocity.^24^ This concept states that the most important parameter in PBM is the total quantity of photons absorbed by the target cells, and it is not important how quickly or how slowly these photons are delivered. This means that $100\;{\mathrm{mW}/\mathrm{cm}}^{2}$ applied for 60 s for a dose of $6\;{J/\mathrm{cm}}^{2}$ will have the same effect as applying $1\;{W/\mathrm{cm}}^{2}$ for 6 s ($6\;{J/\mathrm{cm}}^{2}$) or $6\;{W/\mathrm{cm}}^{2}$ for 1 s ($6\;{J/\mathrm{cm}}^{2}$) using the same spot size.

Numerous studies have shown that, while this law is valuable for many parts of the parameter range, it does not hold true for the entire range.^18^^,^^25^^,^^26^ The previously discussed theories of the biphasic dose response, supported by other studies, are the likely reason for this inaccuracy. Within a certain range of parameters, perhaps between 1 and $100\;{J/\mathrm{cm}}^{2}$, and at power densities from 1 to $100\;{\mathrm{mW}/\mathrm{cm}}^{2}$, this linear reciprocity applies. However, beyond this range, reciprocity does not appear to apply. For instance, there exists a lower threshold (perhaps $0.5\;{\mathrm{mW}/\mathrm{cm}}^{2}$) below which the illumination time could be infinite and would be no different from daylight. Similarly, the upper threshold is fixed by the possible photothermal effect if the power density is too large. The irradiance values, that produce unacceptable heating of the tissue, are governed by the wavelength and are $∼750\;{\mathrm{mW}/\mathrm{cm}}^{2}$ at 800 to 900 nm, about $300\;{\mathrm{mW}/\mathrm{cm}}^{2}$ at 600 to 700 nm, and as low as $100\;{\mathrm{mW}/\mathrm{cm}}^{2}$ at 400 to 500 nm. Furthermore, the illumination time is also important.^17^ There exists a certain minimum length of time (few minutes) that the light needs to be on the tissue for the best effects to occur.^17^

The parameters of most importance in PBM are the power density (irradiance) measured in $\mathrm{mW}/{\mathrm{cm}}^{2}$ and the energy density (fluence) measured in $J/{\mathrm{cm}}^{2}$. Many of the studies discussed here and, indeed, in most of the research literature, are based on the inaccurate statement of the laser output in Watts. Depending on the area irradiated by this beam of photons, the power density and the cellular effects produced will be very different.

As an example, 1 W delivered through a 400  μm diameter optical fiber will produce a power density of $796\;{W/\mathrm{cm}}^{2}$ while the same 1 W delivered through an 8-mm diameter therapy hand-piece will produce a power density of only $2\;{W/\mathrm{cm}}^{2}$.

Energy density is frequently reported in research literature but the spot area at the tissue is often omitted. This error makes it impossible to verify their findings or to see how they calculated the vital energy density information. Inconsistency in reporting these parameters is a major source of contradictory research findings and has done much to hinder the acceptance of PBM effects.

Another important factor that must be taken into account is the optical properties of the tissue itself.^27^ Since the light is generally delivered as a surface spot shone onto the skin, the number of photons that actually penetrate into the tissue to arrive at the pathological lesion is highly variable.^28^ The first issue to be addressed is light reflection from the surface of the skin,^29^ which can be minimized if the optical probe is held in firm contact with the skin.^30^ The second issue is scattering of light within tissue. Scattering is wavelength dependent with shorter wavelengths undergoing more intense Mie scattering than longer wavelengths.^31^ The third issue is absorption of the light by chromophores that are not biologically active. These nonactive chromophores are chiefly hemoglobin (both oxyhemoglobin and deoxyhemoglobin), myoglobin, and melanin.^27^ However, it should be noted that some authors have suggested that photodissociation of oxygen from hemoglobin^32^ or NO from myoglobin^33^ could be a relevant mechanism in PBM. There is a growing trend for researchers in PBM to undertake modeling of tissue optical properties either by Monte-Carlo methods^34^ or by use of tissue phantoms.^35^

### 1.1 Mitochondria and Cells

Mitochondria are highly important intracellular organelles whose main function is to act as “power plant” of the cell, generating ATP which is the main source energy for cellular activity and metabolism. Moreover, mitochondria play important roles in regulation of oxidative stress, calcium metabolism, apoptosis, and a host of signaling pathways.^36^ It is believed that mitochondria originated when a primitive eukaryotic cell “captured” a primitive prokaryotic bacterium around the time the “great oxygenation event” occurred on the Earth.^37^

Mitochondria contain the electron transport chain responsible for transferring electrons from NADH through complexes I, II, III, and IV.^38^

When applying light to cells, mitochondria are the initial sites of light absorption and CCO (particularly, the CuA and CuB metal centers) are believed to be the photoacceptors.^39^ Photon absorption results in setting in motion a cascade of reactions known as cellular signaling pathways leading to NO dissociation, ROS production, and increased ATP synthesis.^9^

The number of mitochondria in cells varies widely and it is strongly correlated with the metabolic requirements of the cell (how many chemical reactions the cell has to carry out) and may range from a few to thousands of individual organelles. Cells such as osteoblasts, keratinocytes, and fibroblasts have a lower number of mitochondria, whereas muscle cells, neural cells, cells composing internal organs (liver, kidneys, spleen, etc.), and myocardial cells contain a higher number of mitochondria. Broadly speaking, the proportion of mitochondria in a tissue type can be gauged by observing the color of the tissue (without containing any blood). For instance, dark colored tissues (liver, heart, kidney, gray brain matter) have a high concentration of mitochondria since CCO and other cytochromes are the most important cellular pigments, while light colored tissues (skin, bones, tendons) have few mitochondria. The following reports discuss how mitochondrial numbers and mitochondrial activity have been determined in different cells and tissues.^40^^–^^43^

Furthermore, mitochondria in stem cells and induced pluripotent stem cells are poorly developed and low in number; mitochondrial function and structure have even been suggested as indicators of stem cell competence.^44^

The hypothesis of the present review is that the effects of PBM on different tissues can be explained by taking into account two main factors. First, what is the content of mitochondria in the cells comprising the bulk of the tissue? Second, what is the depth? Cells *in vitro* are very superficial, skin and some connective tissues are moderately superficial, while other tissues are deeper, bones, joints, brain, organs, etc. Moreover, tissues with high mitochondrial numbers tend to be deeper than those with low mitochondrial numbers.

Therefore, studies were divided into two groups based on the number of mitochondria at the cellular level and the depth of the tissue level.

Cells of tissues with higher numbers of mitochondria were assembled in one group (brain cells, muscle cells, neural cells, macrophages, monocytes) and cells with fewer mitochondria were assembled in another group (keratinocytes, osteoblasts, chondrocytes, fibroblasts, stem cells). Tissues with abundant mitochondria exist in organs, such as muscle, heart, liver, kidney, cells.

The purpose of this review paper was to compare effective and ineffective studies on cells and tissues in each group. Every effort was made to find or calculate relevant parameters even if they were not explicitly stated in the paper.

## 2 Materials and Methods

This study was conducted following Preferred Reporting Items of Systematic reviews and Meta-analysis.

Research questions: Is it possible to propose a practical protocol of for PBM or LLLT? What are the best parameters that produce a positive result in different circumstances?

### 2.1 Research Strategy for Article Identification

Research was conducted using the following electronic databases: Springer, PubMed, Google Scholar, and Cochrane Database.

Keywords used: LLLT, PBM, LLLT and osseointegration, LLLT and bone graft, LLLT and cells, LLLT and bone regeneration.

After collecting the data, the titles, abstract, and conclusions were checked and unrelated, and obviously biased articles were excluded. Also, all case reports and literature reviews were excluded. Only studies dated from 2007 to 2016 were included.

Evaluations of articles were independently performed by two reviewers. The initial search yielded 250 articles. After exclusion of unrelated articles, only 190 remained. Using the exclusion criteria listed in Table 1 reduced this number to 34 articles.

**Table 1:** Eligibility criteria adapted from Cericato et al.^45^ for selection of the studies.

Reason for exclusion	PubMed	Springer	Google Scholar	Cochrane	Total
Literature and/or systematic review	8	8	11	6	33
Article in language other than English	—	—	15	—	15
Letter from the editor, opinion articles	—	—	8	—	8
Fluence not mentioned	3	2	30	8	43
Use of very high fluence: density greater than $500\;J/{\mathrm{cm}}^{2}$	4	2	8	8	22
Article did not mention power or fluence rate	5	2	16	6	29
Other (book chapter, appendix, bibliograghy, index	—	—	4	2	6
Total exclusion	20	14	92	30	156

### 2.2 Assessment of the Studies

After obtaining full texts of all 34 relevant articles, they were evaluated and scored following the checklist using eligibility criteria adapted from Cericato et al.^45^ described in Table 1. Articles with scores from 0 to 8 points were considered low quality and were excluded. Article with scores from 13 to 15 points were considered high quality while scores from 9 to 12 were considered moderate quality. Table 2 presents the details of the 34 studies finally included in this review.

**Table 2:** Final list of studies that were included together with Cericato score.

Authors	Score (Cericato et al.)^17^
Fernandes et al.^46^	12
Mendez et al.^21^	12
Barbosa et al.^20^	11
Huang et al.^47^	11
Huang et al.^48^	12
Sharma et al.^49^	11
Oron et al.^50^	10
Chen et al.^26^	12
Souza et al.^51^	11
Ferraresi et al.^52^	12
Zhang et al.^53^	12
Wang et al.^54^	12
Amaroli^19^	10
Tschon et al.^55^	11
Pyo et al.^56^	12
Migliario et al.^57^	12
Khadra et al.^32^	11
Skopin et al.^58^	12
Salehpour et al.^59^	11
Wu et al.^58^	12
Lopes-Martins et al.^18^	11
Bozkurt et al.^60^	12
Wang et al.^61^	11
Alves et al.^25^	11
Oron et al.^62^^,^^63^	11
Castano et al.^17^	12
Salehpour et al.^64^	11
Leal junior et al.^65^^,^^66^	12
Ando et al.^13^	11
Zhang et al.^67^	12
Baroni et al.^68^	11
Leal Junior et al.^69^	11
Blanco et al.^70^	12
Disner et al.^71^	12

## 3 Effect of Varying a single parameter on PBM Efficacy

### 3.1 I-Effect of varying wavelength on PBM Efficacy

#### 3.1.1 In vitro studies

It has been shown through many studies that CCO is the most important chromophore that absorbs light. Delpy and Cope^72^ showed that over 50% of the light absorption between 800 and 850 nm was due to cytochrome c oxidase, with hemoglobin (oxy and deoxy) playing a minor role. CCO has two absorption bands, one in the red spectral region (∼660  nm) and another in the NIR spectrum (∼800  nm), which consequently are the wavelengths most often used in PBM^3^.

In their study, Wang et al.^54^ found that the mechanisms of action of 810 and 980 nm laser appeared to have significant differences. While the PBM effect occurred at both wavelengths, the chromophore was different between wavelengths. NIR wavelengths, such as 810 nm, stimulate mitochondrial activity and ATP production. At longer wavelengths, the mechanism of action of 980 nm relies on absorption by water leading to the activation of heat (or light)-gated ion channels and promotes cell proliferation via the TRPV1 calcium ion channel pathway.

The same study compared the effect on stem cell differentiation of these two different wavelengths, 810 and 980 nm. For each wavelength, different doses were used from 0.03 to $10\;{J/\mathrm{cm}}^{2}$, spot size $4\;{\mathrm{cm}}^{2}$, irradiance $16\;{\mathrm{mW}/\mathrm{cm}}^{2}$, power 64 mW, and time of irradiance ($3\;{J/\mathrm{cm}}^{2}$, 188 s) and ($0.3\;{J/\mathrm{cm}}^{2}$, 18.8 s). The irradiance was adjusted by varying the distance between the laser and the target cells.

Both wavelengths showed a biphasic dose response. At 980 nm, a peak dose response was seen at 0.03 and $0.3\;{J/\mathrm{cm}}^{2}$ while 810 nm showed a peak response at $3\;{J/\mathrm{cm}}^{2}$. Moreover, the dose of $0.3\;{J/\mathrm{cm}}^{2}$ with the 980-nm laser had a better effect than any of the other groups.

A second study by Wang compared the effects of delivering four different wavelengths (420, 540, 660, and 810 nm) using the same parameters of $3\;{J/\mathrm{cm}}^{2}$ at $16\;{\mathrm{mW}/\mathrm{cm}}^{2}$, on human adipose-derived stem cell differentiation into osteoblasts. They found that 420- and 540-nm wavelengths were more effective in stimulating osteoblast differentiation compared to 660 and 810 nm. Intracellular calcium was higher after 420 and 540 nm and could be inhibited by the TRP channel inhibitors, capsazepine and SKF96365. They concluded that using blue and green wavelengths activated the light-gated calcium channels rather than CCO.^61^^,^^73^

#### 3.1.2 In vivo studies

Mendez et al.^21^ compared, histologically, the effect of using two different wavelengths (GaAlAs 830 nm and InGaAl 685 nm) on repair of cutaneous wounds in rats. The control group received no treatment; group II was irradiated with 685 nm, using a fluence of $20\;{J/\mathrm{cm}}^{2}$ with a spot diameter of 0.6 mm; group III was irradiated using 830 nm, $20\;{J/\mathrm{cm}}^{2}$; group IV was irradiated with both 830 and 685 nm using a total of $20\;{J/\mathrm{cm}}^{2}$; group V with 830 nm using $50\;{J/\mathrm{cm}}^{2}$; group VI with 685 nm, $50\;{J/\mathrm{cm}}^{2}$ and group VII using 830 and 685 nm, $50\;{J/\mathrm{cm}}^{2}$. Laser therapy was repeated four times over 7 days at 48 h intervals. They concluded that better results were observed when combining both wavelengths of 830 and 685 nm and attributed this advantage to different absorption and penetration. When comparing the two wavelengths used separately, 830 nm showed better results. While combining the wavelengths provides valuable information, it was not appropriate to include it in the tables of effectiveness.

Barbosa et al.^20^ compared the effect of light application on bone healing in rats using red and infrared wavelengths. Forty-five rats were divided into three groups after femoral osteotomy: Gr I was used as control; Gr II was submitted to laser treatment using a red wavelength (660 to 690 nm); and Gr III were treated using an infrared laser (790 to 830 nm). Laser therapy was applied immediately after osteotomy and repeated every 48 h, three times a week, for a total of nine sessions over 21 days. The output power was set at 100 mW, energy 4 J, spot size $0.028\;{\mathrm{cm}}^{2}$, power density $3.5\;{W/\mathrm{cm}}^{2}$ for 40 s producing a fluence of $140\;{J/\mathrm{cm}}^{2}$. Animals were sacrificed, the femurs removed and subjected to optical densitometry analysis after 7, 14, and 21 days (five per group).^20^ After 7 days, both laser-treated groups had significantly higher mean bone optical density compared with the control group but no significant difference between the two laser groups was seen. After 14 days, only Gr III treated with infrared energy showed significantly higher bone density than the control group. After 21 days, no significant difference of the mean bone density between the three groups was seen. They concluded that PBM accelerated bone repair in the initial phase and suggested that PBM in bone repair is both timing and wavelength dependent.

Al-Watban and Zhang^16^ compared the efficacy of accelerating wound healing in diabetic rats using visible and NIR diode lasers at wavelengths of: 532, 633, 670, 810, and 980 nm. Each wavelength was delivered at doses of 5, 10, 20 and $30\;{J/\mathrm{cm}}^{2}$, using the same power density for all the wavelength of $22\;{\mathrm{mW}/\mathrm{cm}}^{2}$ except for 633 nm (irradiance used: $15.5\;{\mathrm{mW}/\mathrm{cm}}^{2}$) and 532 nm ($10\;{\mathrm{mW}/\mathrm{cm}}^{2}$). Results showed that there was a significant difference between the NIR and visible wavelengths with visible wavelengths being more effective than NIR. They also concluded that the optimum wavelength was 633 nm and the optimum dose was $10\;{J/\mathrm{cm}}^{2}$.

These studies suggest that the relationship between wavelength and fluence is crucial. If the target is CCO, it is well accepted that red light (630 to 670 nm) or near-infrared light (780 to 940 nm) will have positive effects, using fluences in the stimulatory range of 3 to $10\;{J/\mathrm{cm}}^{2}$.^16^

However, if the desired chromophore is ion channels within cells, the wavelengths that best affect the calcium channels are in the range of 420 to 540 nm.^54^^,^^61^ Delivering just $3\;{J/\mathrm{cm}}^{2}$ when using $16\;{\mathrm{mW}/\mathrm{cm}}^{2}$ will have the best effect. Using the higher wavelength of 980 nm may also have a beneficial effect for targeting water as a chromophore.^54^

Disner et al.^71^ studied the effect of PBMT delivered to the head (over right prefrontal cortex) combined with attention bias modification (ABM) therapy on 51 human patients with elevated symptom of depression. PBMT was administered before and after blocks of ABM using 1064 nm, 3.4 W, irradiance of $250\;{\mathrm{mW}/\mathrm{cm}}^{2}$ ($3,400\;\mathrm{mW}/13.6\;{\mathrm{cm}}^{2}=250\;{\mathrm{mW}/\mathrm{cm}}^{2}$) for 4 min and a cumulative fluence of $60\;{J/\mathrm{cm}}^{2}$ ($0.25\;{W/\mathrm{cm}}^{2}\times 240\;s=60\;{J/\mathrm{cm}}^{2}$). They found that PBMT led to greater symptom improvement especially among participants, whose attention span was responsive to ABM, and they concluded that the beneficial effect of ABM could be improved by adjunctive interventions, such as right prefrontal PBMT.

### 3.2 II-Effect of Varying Energy Density and Power Density on PBM Efficiency

#### 3.2.1 In vitro studies with cells with high number of mitochondria

Fernandez et al.^46^ stimulated the M1 profile (macrophages can have two different phenotypes called M1 and M2 depending on the type of cytokines they produce) of macrophages by using two different sets of laser parameters: 660 nm, 15 mW, $0.375\;{W/\mathrm{cm}}^{2}$, 20 s for $7.5\;{J/\mathrm{cm}}^{2}$ and 780 nm, 70 mW, $1.75\;{W/\mathrm{cm}}^{2}$, 1.5 s for $2.6\;{J/\mathrm{cm}}^{2}$ (the spot area calculated by current authors from available information was $0.04\;{\mathrm{cm}}^{2}$). Results showed that both lasers were able to decrease TNFα and iNOS expression but parameters used for 780 nm gave an additional decrease. Also, parameters used for 660 nm gave an up-regulation of IL-6 expression and production. They concluded that using 780 nm with high power and low energy density or 660 nm with low power and high energy density achieved similar results and the additional decrease by the parameters used with 780 nm suggest that this wavelength returned the cells to a nonstimulated state.

Lopes-Martins et al.^74^ found a true biphasic response occurred in the neutrophils isolated from mice treated with different energy densities (1, 2.5, and $5\;{J/\mathrm{cm}}^{2}$) with a maximum effect at $2.5\;{J/\mathrm{cm}}^{2}$.

Huang et al.^47^ irradiated cortical neuronal cells with a diode laser using 810 nm, $20\;{\mathrm{mW}/\mathrm{cm}}^{2}$, $3\;{J/\mathrm{cm}}^{2}$, spot size of 5 cm, 150 s. They found that laser treatment reduced oxidative stress in primary cortical neurons *in vitro*.

Studies using PBM *in vitro* on cells with high numbers of mitochondria that reported positive results are summarized in Table 3. Ineffective parameters *in vitro* in cells with high numbers of mitochondria are reported in Table 7. In some cases, the same studies are included in both Tables 3 and 7 (effective and ineffective parameters) when the authors varied the parameters.

**Table 3:** Effective treatment of PBM: *in vitro* studies in cells with higher number of mitochondria.

Authors	Wavelength (nm)	Fluence	Irradiance	Cell type
Fernandes et al.^46^	780	$2.6\;{J/\mathrm{cm}}^{2}$	$1.75\;{W/\mathrm{cm}}^{2}$; 70 mW, $0.04\;{\mathrm{cm}}^{2}$, 1.5 s	Macrophage
Huang et al.^47^	810	$3\;{J/\mathrm{cm}}^{2}$	$20\;{\mathrm{mW}/\mathrm{cm}}^{2}$; 150 s, spot size 5 cm	Neural cells
Huang et al.^48^	810	$3\;{J/\mathrm{cm}}^{2}$	$25\;{\mathrm{mW}/\mathrm{cm}}^{2}$, 2 min, spot size 5 cm	Neural cells
Sharma et al.^49^	810	0.03, 0.3, 3, 10, peak at $3\;{J/\mathrm{cm}}^{2}$	$25\;{\mathrm{mW}/\mathrm{cm}}^{2}$	Mouse cortical neuron
Oron et al.^50^	808	$0.05\;{J/\mathrm{cm}}^{2}$	$50\;{\mathrm{mW}/\mathrm{cm}}^{2}$	Human neural cells
Chen et al.^26^	808	$1\;{J/\mathrm{cm}}^{2}$	$44.7\;{\mathrm{mW}/\mathrm{cm}}^{2}$ 170 mW, $3.8\;{\mathrm{cm}}^{2}$, 22.4 s	Monocyte
Souza et al.^51^	780	$3\;{J/\mathrm{cm}}^{2}$	$275\;{\mathrm{mW}/\mathrm{cm}}^{2}$ [Power=70 mW, 1.5 s (2×) effective power 53.9 mW] $\text{Area}=0.196\;{\mathrm{cm}}^{2}$ $\text{Beam spot area}=0.04\;{\mathrm{cm}}^{2}$	Macrophage
Ferraresi et al.^52^	Cluster 40 LEDs (20 infrared 850 nm and 20 red 630 nm)	$2.5\;{J/\mathrm{cm}}^{2}$	$28\;{\mathrm{mW}/\mathrm{cm}}^{2}$ 50 mW (IR) and 25 mW (red) Cluster: 1000 mW (IR) and 500 mW (red) $45\;{\mathrm{cm}}^{2}$, 90 s, distance: 156 mm	Myotube C2C12
Amaroli et al.^19^	808	$3.0\;{J/\mathrm{cm}}^{2}$	$100\;{\mathrm{mW}/\mathrm{cm}}^{2}$ 100 mW $\text{spot area}:1\;{\mathrm{cm}}^{2}$	Paramecium
Amaroli^19^	808	$64\;{J/\mathrm{cm}}^{2}$	$1000\;{\mathrm{mW}/\mathrm{cm}}^{2}$ 100 mW, $\text{spot area}=1\;{\mathrm{cm}}^{2}$	Paramecium
Chen et al.^26^	660	$1\;{J/\mathrm{cm}}^{2}$	$0.8\;{\mathrm{mW}/\mathrm{cm}}^{2}$ 6 mW, $7.5\;{\mathrm{cm}}^{2}$ 1250 s	Monocyte
Chen et al.^26^	660	$2\;{J/\mathrm{cm}}^{2}$	$0.8\;{\mathrm{mW}/\mathrm{cm}}^{2}$ 6 mW, $7.5\;{\mathrm{cm}}^{2}$ 2500 s	Monocyte
Souza et al.^51^	660	$7.5\;{J/\mathrm{cm}}^{2}$ effective fluence $1.15\;{J/\mathrm{cm}}^{2}$	$57.4\;{\mathrm{mW}/\mathrm{cm}}^{2}$ Power=15 mW, 20 s Effective power 11.25 mW $\text{Irradiated area}=0.196\;{\mathrm{cm}}^{2}$ $\text{Beam spot area}=0.04\;{\mathrm{cm}}^{2}$	Macrophage
Fernandez et al.^46^	660	$7.5\;J/{\mathrm{cm}}^{2}$	$0.375\;{W/\mathrm{cm}}^{2}$ 15 mW, $0.04\;{\mathrm{cm}}^{2}$, 20 s	Macrophage

#### 3.2.2 In vitro studies with cells with lower numbers of mitochondria

Tschon et al.^55^ irradiated osteoblast–like cells using a 915-nm diode laser at the following parameters: 100 Hz pulsed mode, 50% duty cycle, and output power of 0.575 W. Laser energy was delivered in defocused mode using a concave lens to cover the growth area ($1.91\;{\mathrm{cm}}^{2}$) at distance of 19 mm (power density calculated by current authors from available information was $150\;{\mathrm{mW}/\mathrm{cm}}^{2}$). The laser was applied for 48, 96, and 144 s producing doses of 5, 10, and $15\;{J/\mathrm{cm}}^{2}$ (energy density calculated by current authors from available information was 7.2, 14.4, and $21.56\;{\mathrm{mJ}/\mathrm{cm}}^{2}$), and specimens were examined after 4, 24, 48, and 72 h. *In vitro* scratch wounds treated with 5 and $10\;{J/\mathrm{cm}}^{2}$ were the first to reach complete coverage after 72 h, followed by $15\;{J/\mathrm{cm}}^{2}$, which reached complete healing after 96 h.

Pyo et al.^56^ studied the effect of hypoxia and PBM on the expression of bone morphogenetic protein-2 (BMP-2); transforming growth factor-beta-1 (TGF-β1); type I collagen, osteocalcin; hypoxia inducible factor-1 (HIF-1) and AKT. Osteoblast cells were cultured under 1% oxygen tension and then exposed to hypoxia. These cells were then irradiated with an 808 nm diode laser; 1000 mW, continuous wave (CW) for 15 s for a stated energy density of $1.2\;{J/\mathrm{cm}}^{2}$ at each session (power density calculated by current authors from available information was $80\;{\mathrm{mW}/\mathrm{cm}}^{2}$). Other cells were cultured 24 h more under hypoxia and irradiated a second and third time for a total energy density of 1.2, 2.4, and $3.6\;{J/\mathrm{cm}}^{2}$. Finally, further hypoxia was applied to the cells after irradiation. Cells were not exposed to laser energy in the control groups and were incubated under hypoxia at 1, 24, and 48 h. Results showed that hypoxia did not affect osteoblast viability (in the control group) and BMP-2, but it resulted in a decrease in osteocalcin, TGF-β, and expression of type I collagen. However, PBM applied to hypoxic osteoblasts stimulated osteoblast differentiation and proliferation through an increased expression of BMP-2, osteocalcin, and TGF-β. In addition, PBM inhibited HIF-1 expression and inhibited production of Akt.

Migliario et al.^57^ irradiated murine preosteoblasts (MC-3 T3 –E1) in order to evaluate the effect of PBM on ROS in cells labeled with an ROS marker. They used a diode laser at 930 nm, 1 W, irradiation time of 1, 5, 10, 25, and 50 s, for a delivered fluence of 1.57, 7.87, 15.74, 39.37, and $78.75\;{J/\mathrm{cm}}^{2}$ (spot area calculated by current authors from available information was $0.63\;{\mathrm{cm}}^{2}$ and irradiance of $1.57\;{W/\mathrm{cm}}^{2}$). The laser application was delivered three times at 0, 24, and 48 h. They found that ROS generation was dose dependent and doubled at higher fluences (25 to $50\;{J/\mathrm{cm}}^{2}$). Also, laser irradiation was able to increase preosteoblast proliferation starting from a fluence of $5\;{J/\mathrm{cm}}^{2}$. Increasing the fluence produced an increase in cell proliferation up to $25\;{J/\mathrm{cm}}^{2}$ and then a decrease at $50\;{J/\mathrm{cm}}^{2}$. The peak of cell proliferation occurred at $10\;{J/\mathrm{cm}}^{2}$. These results are partially in disagreement with other studies that suggest that 1 to $5\;{J/\mathrm{cm}}^{2}$ was optimal for cell proliferation. Contradictory results may be due to differences in irradiation parameters (wavelength, output power, energy density).

Zhang et al.^53^ irradiated fibroblast cells with 628 nm. Power output was constant at 15 mW, irradiance $11.46\;{\mathrm{mW}/\mathrm{cm}}^{2}$, and distance of 0.75 cm. Samples were irradiated for various time periods to yield final energy doses of 0.44, 0.88, 2.00, 4.40, and $9\;{J/\mathrm{cm}}^{2}$. They found a maximum increase in human fibroblast cell proliferation with a fluence of $0.88\;{J/\mathrm{cm}}^{2}$ and a reduction in the proliferation at $9\;{J/\mathrm{cm}}^{2}$.

Khadra et al.^75^ investigated the effect of single and multiple doses on attachment and proliferation of human fibroblasts. Cells were cultured on titanium implants and divided into three groups: group I was used as a control, group II received GaAlAs 830 nm, output power 84 mW, 9 cm distance to the cells, a single dose of $3\;{J/\mathrm{cm}}^{2}$, 360 s (spot area calculated by current authors from available information was $10\;{\mathrm{cm}}^{2}$ and irradiance of $0.0084\;{W/\mathrm{cm}}^{2}$), group III was divided into three subgroups and exposed to multiple doses (one dose on each of three consecutive days) of 0.75, 1.5, and $3\;{J/\mathrm{cm}}^{2}$ corresponding to exposure times of 90, 180, and 360 s (spot area calculated by current authors from available information was $10\;{\mathrm{cm}}^{2}$). Results indicated that samples exposed to multiple doses of 1.5 and $3\;{J/\mathrm{cm}}^{2}$ showed a significantly proliferation. They concluded that the attachment of human fibroblasts to the titanium implant was enhanced by PBM. Both multiple and single doses significantly increased cellular attachment. Finally, $0.75\;{J/\mathrm{cm}}^{2}$ did not promote proliferation and cell attachment.

Skopin and Molitor^58^ studied the effect of using different doses and different irradiances on wound healing in fibroblast cultures using 980-nm diode laser. They applied an irradiance of: 26, 49, 73, 97, and $120\;{\mathrm{mW}/\mathrm{cm}}^{2}$ for a constant 2 min each, delivering 3.1, 5.9, 8.8, 11.6, and $14.4\;{J/\mathrm{cm}}^{2}$. They found a significant increase in cell division when using 26, 73, and $97\;{\mathrm{mW}/\mathrm{cm}}^{2}$. This effect was negated at $120\;{\mathrm{mW}/\mathrm{cm}}^{2}$.

Al-Watban and Andres^76^ studied the effect of He–Ne laser on the proliferation of hamster ovary and human fibroblasts. Irradiance was held constant at $1.25\;{\mathrm{mW}/\mathrm{cm}}^{2}$ using an accumulated dose over three consecutive days of 60 to $600\;{\mathrm{mJ}/\mathrm{cm}}^{2}$. They found a peak response at $180\;{\mathrm{mJ}/\mathrm{cm}}^{2}$. This study suggested that there is activation at a lower dose from $2\;{\mathrm{mJ}/\mathrm{cm}}^{2}$ with a peak at $180\;{\mathrm{mJ}/\mathrm{cm}}^{2}$. At higher doses, greater than $300\;{\mathrm{mJ}/\mathrm{cm}}^{2}$, there was bioinhibition.

Studies using PBM *in vitro* on cells with low numbers of mitochondria that reported positive results are summarized in Table 4. Ineffective parameters *in vitro* in cells with low numbers of mitochondria are reported in Table 8. In some cases, the same studies are included in both Tables 3 and 7 (effective and ineffective parameters) when the authors varied the parameters.

**Table 4:** Effective treatment of PBM: *in vitro* studies in cells with lower number of mitochondria.

Authors	Wavelength (nm)	Fluence ($J/{\mathrm{cm}}^{2}$)	Irradiance	Cell type
Wang et al.^54^	420	3	$16\;{\mathrm{mW}/\mathrm{cm}}^{2}$ $4\;{\mathrm{cm}}^{2}$, 188 s	Adipose stem cells
Wang et al.^54^	540	3	$16\;{\mathrm{mW}/\mathrm{cm}}^{2}$ $4\;{\mathrm{cm}}^{2}$, 188 s	Adipose stem cells
Zhang et al.^53^	628	0.88	$11.46\;{\mathrm{mW}/\mathrm{cm}}^{2}$ Output power 15 mW, 0.76 cm distance to the surface, $\text{area}=9.6\;{\mathrm{cm}}^{2}$	Fibroblast
Zhang et al.^53^	628	2.0	$11.46\;{\mathrm{mW}/\mathrm{cm}}^{2}$ Output power 15 mW, 0.76 cm distance to the surface, $\text{area}=9.6\;{\mathrm{cm}}^{2}$	Fibroblast
Zhang et al.^53^	628	4.4	$11.46\;{\mathrm{mW}/\mathrm{cm}}^{2}$ Output power 15 mW, 0.76 cm distance to the surface, $\text{area}=9.6\;{\mathrm{cm}}^{2}$	Fibroblast
Khadra et al.^32^	830	1.5	$8.4\;{\mathrm{mW}/\mathrm{cm}}^{2}$ 84 mW, $10\;{\mathrm{cm}}^{2}$, 9 cm distance to cells	Fibroblast
Khadra et al.^32^	830	3.0	$8.4\;{\mathrm{mW}/\mathrm{cm}}^{2}$ 84 mW, $10\;{\mathrm{cm}}^{2}$, 360 s, 9 cm distance to cells	Fibroblast
Tschon et al.^55^	915	7.2	$150\;{\mathrm{mW}/\mathrm{cm}}^{2}$, 100 Hz, 50% duty cycle, power 0.575 W, 48 s	Osteoblast
Tschon et al.^55^	915	14.4	$150\;{\mathrm{mW}/\mathrm{cm}}^{2}$ 50% duty cycle, power 0.575 W, 96 s	Osteoblast
Migliario et al.^57^	930	7.8	$1580\;{\mathrm{mW}/\mathrm{cm}}^{2}$ 1 W, 5 s, $0.63\;{\mathrm{cm}}^{2}$	Preosteoblast
Migliario et al.^57^	930	15	$1580\;{\mathrm{mW}/\mathrm{cm}}^{2}$ 1 W, 10 s, $0.63\;{\mathrm{cm}}^{2}$	Preosteoblast
Migliario et al.^57^	930	39	$1580\;{\mathrm{mW}/\mathrm{cm}}^{2}$ 1 W, 25 s, $0.63\;{\mathrm{cm}}^{2}$	Preosteoblast
Pyo et al.^56^	808	1.2	$80\;{\mathrm{mW}/\mathrm{cm}}^{2}$ 15 s, 1 W	Osteoblast
Skopin et al.^58^	980	3.1	$26.7\;{\mathrm{mW}/\mathrm{cm}}^{2}$	Fibroblast
Skopin et al.^58^	980	8.8	$73\;{\mathrm{mW}/\mathrm{cm}}^{2}$	Fibroblast
Skopin et al.^58^	980	11.6	$97\;{\mathrm{mW}/\mathrm{cm}}^{2}$	Fibroblast
Bozkurt et al.^60^	940	18	$0.3\;{W/\mathrm{cm}}^{2}$ 0.3 W, 60 s, distance: 0.5 to 1 mm	Cementoblast
Wang et al.^73^	810	3	$16\;{\mathrm{mW}/\mathrm{cm}}^{2}$ $4\;{\mathrm{cm}}^{2}$, 188 s	Adipose stem cells
Wang et al.^61^	980	0.3	$16\;{\mathrm{mW}/\mathrm{cm}}^{2}$ $4\;{\mathrm{cm}}^{2}$, 18.8 s	Adipose stem cells

#### 3.2.3 In vivo studies in tissues with high number of mitochondria: heart, brain, muscle, inflammation

Oron et al.^62^ treated myocardial infarction with LLLT using an 810-nm laser. Fluence was held constant at $0.9\;{J/\mathrm{cm}}^{2}$ while irradiance was varied to deliver 2.5, 5, and $25\;{\mathrm{mW}/\mathrm{cm}}^{2}$. A peak response was found at $5\;{\mathrm{mW}/\mathrm{cm}}^{2}$, while treatment was less effective when using 2.5 and $25\;{\mathrm{mW}/\mathrm{cm}}^{2}$.

Castano et al.^17^ studied inflammatory arthritis in rats, comparing the effect of using high and low fluences (3 to $30\;{J/\mathrm{cm}}^{2}$) delivered at high and low irradiance (5 to $50\;{\mathrm{mW}/\mathrm{cm}}^{2}$). Effective treatment was observed when using: $30\;{J/\mathrm{cm}}^{2}$ at $50\;{\mathrm{mW}/\mathrm{cm}}^{2}$ for 10 min and $30\;{J/\mathrm{cm}}^{2}$ at $5\;{\mathrm{mW}/\mathrm{cm}}^{2}$ for 100 min. Low fluence of $3\;{J/\mathrm{cm}}^{2}$ at $5\;{\mathrm{mW}/\mathrm{cm}}^{2}$ for 10 min was also effective. Only the dose of $3\;{J/\mathrm{cm}}^{2}$ at $50\;{\mathrm{mW}/\mathrm{cm}}^{2}$ for 1 min was ineffective. They concluded that at higher fluence ($30\;{J/\mathrm{cm}}^{2}$), the PBM effect on arthritis did not depend on irradiance as both high and low irradiance were effective, while at a lower fluence of $3\;{J/\mathrm{cm}}^{2}$, only the lower irradiance was effective. Therefore, they concluded that the duration of the light exposure was of great importance. While some studies found ($3\;{J/\mathrm{cm}}^{2}$, $50\;{\mathrm{mW}/\mathrm{cm}}^{2}$) beneficial, this study did not. They suggest that because the duration was only 1 min, the light did not have sufficient time to produce a sufficient activation of cellular metabolism.^17^

Salehpour et al.^77^ compared the therapeutic effect of a 10-Hz pulsed wave of NIR (810 nm) and red (630 nm) lasers with citalopram in rats that had been subjected to a model of chronic mild stress that causes depression. After inducing stress in rats, they were divided into: group I receiving PBM using NIR 810 nm and group II receiving 630-nm coherent light using identical parameters of: 10-Hz gated wave (50% duty cycle), fluence of $1.2\;{J/\mathrm{cm}}^{2}$ per session, output power 35 and 240 mW, respectively, 2 ms duration for both type of lasers, beam diameter of 3 mm, contact mode, and spot size of $0.07\;{\mathrm{cm}}^{2}$. Laser power was set at 6.2 W in the red wavelength and 39.3 W in the infrared wavelength for an irradiance of 89 and $562\;{\mathrm{mW}/\mathrm{cm}}^{2}$, respectively. The average fluence for each session was $1.2\;{J/\mathrm{cm}}^{2}$ and totaling $14.4\;{J/\mathrm{cm}}^{2}$ for the entire 12 session treatment. Finally, group III was treated with the antidepressant drug citalopram that works by decreasing cortisol levels. Results showed that PBM using 10-Hz pulsed NIR laser had a better effect than red laser and the same effect as citalopram.

Salehpour et al.^59^ studied brain mitochondrial function in mice after inducing mitochondrial dysfunction by administration of D-galactose. This model is considered to be a model of age-related cognitive dysfunction. Animals were treated with wavelengths of 660 and 810 nm at two different fluences: 4 and $8\;{J/\mathrm{cm}}^{2}$, 10 Hz, $4.75\;{W/\mathrm{cm}}^{2}$, 88% duty cycle, 200 mW, in contact, three times a week, 48 h between sessions, and 7-mm diameter power meter sensor. They found poor results with both wavelengths at $4\;{J/\mathrm{cm}}^{2}$ and an amelioration of the aging-induced mitochondrial dysfunction with $8\;{J/\mathrm{cm}}^{2}$

Wu et al.^78^ induced traumatic brain injury (TBI) in mice and treated the animals using 660, 730, 810, or 980 nm, single dose treatment of $36\;{J/\mathrm{cm}}^{2}$ using an irradiance of $15\;{\mathrm{mW}/\mathrm{cm}}^{2}$, 4-min duration, 4 h after injury. They found a significant improvement for mice having moderate to severe injury only when using 660 nm and 810 nm. The most desirable effect was seen at 810 nm, and both 730 and 980 nm did not produce any benefit.

Lopes-Martins et al.^18^ investigated the effect of PBM on muscular fatigue in rats during tetanic contractions. Four groups of 32 rats received different doses of PBMT (0.5, 1.0, and $2.5\;{J/\mathrm{cm}}^{2}$), using parameters of 655 nm, spot area $0.08\;{\mathrm{cm}}^{2}$, 25 mW, 2.5 mW; $31.25\;{\mathrm{mW}/\mathrm{cm}}^{2}$. Groups: $0.5\;{J/\mathrm{cm}}^{2}$ (32 s), $1\;{J/\mathrm{cm}}^{2}$ (80 s), $2.5\;{J/\mathrm{cm}}^{2}$ (160 s). Only the groups of 0.5 and $1\;{J/\mathrm{cm}}^{2}$ prevented the development of muscular fatigue in rats during repeated tetanic contractions.

Lopes-Martins et al.^74^ in another study used 650-nm wavelength on acute inflammatory pleurisy in mice. Using the same power of 2.5 mW but different fluences of 3, 7.5, and $15\;{J/\mathrm{cm}}^{2}$. They found that under these conditions, $7.5\;{J/\mathrm{cm}}^{2}$ were more effective than either 3 or $15\;{J/\mathrm{cm}}^{2}$.

De Almeida et al.^79^ studied muscle performance after inducing muscle contraction in 30 rats. Using 904 nm, 15-mW average power and different energies (0.1, 0.3, 1.0, and 3.0 J) they found that the 1.0 and 3.0 J groups showed significant enhancement (P<0.01) in total work. They conclude that 1.0 J decreased postexercise muscle damage and enhanced muscle performance.

Studies using PBM *in vivo* in tissues with high numbers of mitochondria that reported positive results are summarized in Table 5. Ineffective parameters PBM *in vivo* in tissues with high numbers of mitochondria are reported in Table 9. In some cases, the same studies are included in both Tables 5 and 9 (effective and ineffective parameters) when the authors varied the parameters.

**Table 5:** Effective PBM treatment: *in vivo* on tissues with higher number of mitochondria.

Authors	Wavelength (nm)	Fluence	Irradiance	Tissue type
Alves et al.^25^	808	$142.4\;J/{\mathrm{cm}}^{2}$	$1.78\;{W/\mathrm{cm}}^{2}$ 4 J, 50 mW, $0.028\;{\mathrm{cm}}^{2}$, 80 s per point	Arthritis
Oron et al.^62^^,^^63^	810	$0.3\;J/{\mathrm{cm}}^{2}$	$5\;{\mathrm{mW}/\mathrm{cm}}^{2}$ 5 mW, area $1.1\;{\mathrm{cm}}^{2}$, 60 s	Heart
Oron et al.^62^^,^^63^	810	$0.9\;J/{\mathrm{cm}}^{2}$	$5\;\mathrm{mW}/{\mathrm{cm}}^{2}$	Myocardium tissue
Castano et al.^17^	810	$30\;J/{\mathrm{cm}}^{2}$	$50\;\mathrm{mW}/{\mathrm{cm}}^{2}$	Arthritis
Castano et al.^17^	810	$30\;J/{\mathrm{cm}}^{2}$	$5\;\mathrm{mW}/{\mathrm{cm}}^{2}$	Arthritis
Castano et al.^17^	810	$3\;J/{\mathrm{cm}}^{2}$	$5\;\mathrm{mW}/{\mathrm{cm}}^{2}$	Arthritis
Salehpour et al.^59^	810	$1.2\;J/{\mathrm{cm}}^{2}$	$560\;{\mathrm{mW}/\mathrm{cm}}^{2}$ 39.3 W, spot size $0.07\;{\mathrm{cm}}^{2}$	Brain
Salehpour et al.^64^	810	$8\;J/{\mathrm{cm}}^{2}$	$89\;{\mathrm{mW}/\mathrm{cm}}^{2}$ 6.2 W, spot size $0.07\;{\mathrm{cm}}^{2}$	Brain
Wu et al.^78^	810	$36\;J/{\mathrm{cm}}^{2}$	$15\;{\mathrm{mW}/\mathrm{cm}}^{2}$	Brain
Blanco et al.^70^	1064	$250\;\mathrm{mW}/{\mathrm{cm}}^{2}$	$60\;{J/\mathrm{cm}}^{2}$	Brain (human)
Disner et al.^71^	1064	$250\;\mathrm{mW}/{\mathrm{cm}}^{2}$	$60\;J/{\mathrm{cm}}^{2}$	Brain (human)
Ando et al.^13^	810	$36\;J/{\mathrm{cm}}^{2}$	$50\;\mathrm{mW}/{\mathrm{cm}}^{2}$	TBI
Zhang et al.^67^	810	Fluence reaching the cortex 1.8 to $2.5\;{J/\mathrm{cm}}^{2}$ Average irradiance $36\;{J/\mathrm{cm}}^{2}$	$150\;{\mathrm{mW}/\mathrm{cm}}^{2}$ Pulse freq 10 Hz, pulse duration 50 ms, 4 min	TBI
Salehpour et al.^68^	810	$1.2\;J/{\mathrm{cm}}^{2}$	89 and $562\;{\mathrm{mW}/\mathrm{cm}}^{2}$ 35 and 240 mW 10 Hz, 50% duty cycle; $0.07\;{\mathrm{cm}}^{2}$	Brain
Baroni et al.^68^	Cluster with 69 LEDs 660/850 nm	$206.89\;J/{\mathrm{cm}}^{2}$	$6.89\;{W/\mathrm{cm}}^{2}$ 200 mW; 6 J per diode (30 s); $0.02\;{\mathrm{cm}}^{2}$ 30 J per application point (5×6 J)6 application points: total energy 180 J	Femoral quadriceps
Zhang et al.^80^	635	$0.96\;J/{\mathrm{cm}}^{2}$	$6.37\;{\mathrm{mW}/\mathrm{cm}}^{2}$ 5 mW, laser beam width 10 mm, 150 s	Preconditioning myocardium
Salehpour et al.^59^	660	$8\;J/{\mathrm{cm}}^{2}$	$4.75\;{W/\mathrm{cm}}^{2}$ 88% duty cycle, 200 mW, in contact three times a week, 7 mm diameter	Brain
Wu et al.^78^	660	$36\;J/{\mathrm{cm}}^{2}$	$15\;{\mathrm{mW}/\mathrm{cm}}^{2}$	Brain
Lopes-Martins et al.^18^	655	$0.5\;J/{\mathrm{cm}}^{2}$	$31.25\;{\mathrm{mW}/\mathrm{cm}}^{2}$ 2.5 mW, spot area $0.08\;{\mathrm{cm}}^{2}$, 25 mW, 32 s	Muscle
Lopes-Martins et al.^18^	655	$1\;J/{\mathrm{cm}}^{2}$	$31.25\;{\mathrm{mW}/\mathrm{cm}}^{2}$ 2.5 mW, spot area $0.08\;{\mathrm{cm}}^{2}$, 25 mW, 80 s, 2.5 mW	Muscle

#### 3.2.4 In vivo studies in tissues with a lower number of mitochondria: skin, bone, cartilage

Lanzafame et al.^15^ treated pressure ulcers in mice with a 670-nm diode laser. Maintaining a constant fluence of $5\;{J/\mathrm{cm}}^{2}$ and using different irradiances (0.7, 2, 8, $40\;{\mathrm{mW}/\mathrm{cm}}^{2}$), they found a significant improvement at $8\;{\mathrm{mW}/\mathrm{cm}}^{2}$.

Prabhu et al.^81^ found a biphasic dose response on excisional wound healing in mice when using a He–Ne laser (632 nm, 7 mW, $4\;{\mathrm{mW}/\mathrm{cm}}^{2}$ at different fluences (1, 2, 3, 4, 6, 8, and $10\;{J/\mathrm{cm}}^{2}$). A clear biphasic dose response occurred with a peak benefit at a fluence of $2\;{J/\mathrm{cm}}^{2}$ and an inhibitory effect at the higher dose of $10\;{J/\mathrm{cm}}^{2}$.

Gal et al.^82^ compared the wound tensile strength in rats at different power densities using 670 nm. A positive effect was seen when using $4\;{\mathrm{mW}/\mathrm{cm}}^{2}$ delivered for 20 min, 50 s, ($5\;{J/\mathrm{cm}}^{2}$), but this effect was not seen when using $15\;{\mathrm{mW}/\mathrm{cm}}^{2}$ delivered for 5 min, 33 s, ($5\;{J/\mathrm{cm}}^{2}$) at the same wavelength. This suggests that delivering the same fluence at a lower irradiance over more time was more effective.

Al-Watban and Delgado^83^ studied, *in vivo*, the effect of laser irradiation on burn wound healing in rats. They created a superficial burn with an area of $1.534\;{\mathrm{cm}}^{2}$ and irradiated the wound with a diode laser at 670 nm, 200 mW, three times per week for 12 weeks at different doses of 1, 5, 9, and $19\;{J/\mathrm{cm}}^{2}$. Only the groups receiving the lower doses of 1 and $5\;{J/\mathrm{cm}}^{2}$ showed significantly better wound healing compared to the control, with the greatest effect obtained at $1\;{J/\mathrm{cm}}^{2}$.

Studies using PBM *in vivo* in tissues with low numbers of mitochondria that reported positive results are summarized in Table 6. Ineffective parameters PBM *in vivo* in tissues with low numbers of mitochondria are reported in Table 10. In some cases, the same studies are included in both Tables 6 and 10 (effective and ineffective parameters) when the authors varied the parameters.

**Table 6:** Effective PBM treatment: *in vivo* on tissues with lower number of mitochondria.

Authors	Wavelength (nm)	Fluence ($J/{\mathrm{cm}}^{2}$)	Irradiance	Tissue type
Mendez et al.^21^	830	50	$125\;{\mathrm{mW}/\mathrm{cm}}^{2}$ 35 mW 0.6 cm diameter	Wound healing
Lanzarfane et al.^15^	670	5	$8\;\mathrm{mw}/{\mathrm{cm}}^{2}$	Ulcer
Prabhu et al.^21^	632	2	$4.0\;\mathrm{mW}/{\mathrm{cm}}^{2}$ 7 mw, $1.75\;{\mathrm{cm}}^{2}$	Wound healing
Gal et al.^21^	670	5	$15\;\mathrm{mw}/{\mathrm{cm}}^{2}$	Wound tensile strength
Al-Watban et al.^21^	670	1 and 5	$130\;{\mathrm{mW}/\mathrm{cm}}^{2}$ 200 mW, $1.534\;{\mathrm{cm}}^{2}$	Wound healing
Mendez et al.^21^	830	20	$125\;{\mathrm{mW}/\mathrm{cm}}^{2}$ 35 mW, 0.6 cm diameter	Wound healing
Barbosa et al.^20^	790	140	$3500\;{\mathrm{mW}/\mathrm{cm}}^{2}$ 100 mW, 4 J, spot size $0.028\;{\mathrm{cm}}^{2}$	Bone
Barbosa et al.^20^	830	140	$3500\;{\mathrm{mW}/\mathrm{cm}}^{2}$ 100 mW, 4 J, spot size $0.028\;{\mathrm{cm}}^{2}$	Bone

### 3.3 III-Effect of Varying the Mode of Delivery on PBM Efficiency: CW or Pulsed

In a comprehensive literature review,^84^ Hamblin included 33 studies, nine of them directly comparing pulsed wave and CW. Six of these studies found that pulsed wave offered better results than CW; one study found that both modes were equally effective and only two studies reported better result using CW. Hamblin et al. concluded from this review that pulsed light may be superior to CW light, particularly for wound healing and poststroke management, whereas CW may be more beneficial in patients requiring nerve regeneration. In addition, they concluded that it is impossible to draw any correlation between pulse frequency and pathological condition. They found that no particular frequency appears to be more or less effective than others. Finally, this review reported that the following frequencies were beneficial: 2, 10, 25, 50 Hz when using (670 nm, 20 mW, energy density, $2\;{J/\mathrm{cm}}^{2}$), 100 Hz when using (808 nm, $37.5\;{\mathrm{mW}/\mathrm{cm}}^{2}$, $0.9\;{J/\mathrm{cm}}^{2}$) 292 Hz when using ($800\;{\mathrm{mW}/\mathrm{cm}}^{2}$; $21.6\;{J/\mathrm{cm}}^{2}$), 600 Hz when using (670 nm, 10 mW, $5\;{J/\mathrm{cm}}^{2}$), 1000 Hz when using (808 nm, $7.5\;{\mathrm{mW}/\mathrm{cm}}^{2}$, 0.9–1.2 J, duty cycle, 30%), 1500 Hz when using ($5\;{\mathrm{mW}/\mathrm{cm}}^{2}$); 3000 Hz when using ($10\;{\mathrm{mW}/\mathrm{cm}}^{2}$) and 8000 Hz (N/A).

Gigo-Benato et al.^14^ compared the effect of combined CW and pulsed laser (CW+PW) using 808 nm (CW) and 905 nm (PW) to either the CW (808 nm) or PW (905 nm) laser used separately. CW was applied at $29\;{J/\mathrm{cm}}^{2}$ while the pulsed wave laser was applied at $40\;{J/\mathrm{cm}}^{2}$. Results suggested that the combined laser was more effective in nerve regeneration than the CW alone or the PW alone.

Al-Watban and Zhang^16^ evaluated the effects of using both pulsed and CW PBM in rats wound healing. After creation of elliptical wounds, animals were treated with a 635-nm diode laser, average power of 3.4 mW, spot size of $3.8\;{\mathrm{cm}}^{2}$, wound size of $1.04\;{\mathrm{cm}}^{2}$, irradiance of $0.89\;{\mathrm{mW}/\mathrm{cm}}^{2}$, treatment duration 18.7 min and fluence of $1\;{J/\mathrm{cm}}^{2}$, three times per week. The dose was delivered using either CW or pulsed mode at: 100, 200, 300, 400, or 500 Hz. They found that the effect of using CW was more efficient than using pulsed laser and, when comparing different frequencies, 100 Hz had better effect on wound healing than the other frequencies.

This article contradicts Hamblin, who concluded that pulsed mode was more effective than CW in wound healing. Perhaps, Al-Watban found that CW was more efficient because he did not use the same fluence in CW that he used in pulsed mode. Moreover, he used gated CW rather than true pulsed wave.^16^

Ando et al.^13^ treated TBI in mice comparing pulsed and CW 810-nm laser irradiation. The parameters used were: 810-nm diode laser, irradiance of $50\;{\mathrm{mW}/\mathrm{cm}}^{2}$, spot diameter of 1 cm onto the injured head with a 12-min exposure giving a fluence of $36\;{J/\mathrm{cm}}^{2}$. They found that 10 Hz produced better results than 100 Hz or continuous mode.

el Sayed and Dyson^85^ compared the effect of four different frequencies (2.5, 20, 292 and 20,000 Hz) and found that only 20 and 292 Hz were beneficial.

Sushko et al.^86^ investigated pain induced in mice by hypodermic injection of 20 ml of 5% formalin solution into the footpad. They irradiated the mice using 640 and 880 nm LED in continuous or pulsed mode for 10 min. They found that pulsed mode was more effective than CW and frequencies of 10 and 8000 Hz were most effective, whereas pulse repetition rates of 200 and 600 Hz were less effective.

Ueda and Shimizu^87^ studied the effect of three different pulse repetition rates on osteoblast-like cells from rats using these parameters (830 nm, 500 mW, 0.48 to $3.84\;{J/\mathrm{cm}}^{2}$) in CW mode and (1, 2, and 8 Hz) in pulsed mode. They found that 1 and 2 Hz markedly stimulated cellular proliferation, ALP activity, ALP gene expression, and bone nodule formation, and that 2 Hz was the best pulse repetition rate to stimulate bone nodule formation.

## 4 Review of Which Parameters Lead to Effective and Ineffective PBMT

It is difficult to compare studies done with different parameters, protocols, treatment objectives, and biological target tissues. Often, parameters are not completely presented or are of questionable accuracy. In this part of the review analysis, an attempt is made to draw at least some general inferences from the data presented in Tables 3–10.

**Table 7:** Ineffective treatment of PBM: *in vitro* studies in cells with higher number of mitochondria.

Authors	Wavelength (nm)	Fluence ($J/{\mathrm{cm}}^{2}$)	Irradiance	Cell type
Sharma et al.^49^	810	30	$25\;{\mathrm{mW}/\mathrm{cm}}^{2}$	Mouse cortical neurons
Chen et al.^26^	660	3	$0.8\;{\mathrm{mW}/\mathrm{cm}}^{2}$ 6 mW, $7.5\;{\mathrm{cm}}^{2}$ 3750 s	Monocyte
Chen et al.^26^	660	2	$0.8\;{\mathrm{mW}/\mathrm{cm}}^{2}$ 6 mW, $7.5\;{\mathrm{cm}}^{2}$ 2500 s	Monocyte
Amaroli et al.^19^	808	3.0	$1000\;{\mathrm{mW}/\mathrm{cm}}^{2}$, 1 W, $1\;{\mathrm{cm}}^{2}$ spot area	Paramecium
Amaroli et al.^19^	808	64	$100\;{\mathrm{mW}/\mathrm{cm}}^{2}$, 1 W, $1\;{\mathrm{cm}}^{2}$ spot area	Paramecium

**Table 8:** Ineffective treatment of PBM *in vitro* studies in cells with lower number of mitochondria.

Authors	Wavelength (nm)	Fluence ($J/{\mathrm{cm}}^{2}$)	Irradiance	Cell type
Tschon et al.^55^	915	20.56	$150\;{\mathrm{mW}/\mathrm{cm}}^{2}$ 100 Hz, 50% duty cycle, power 0.575 W 144 s	Osteoblast
Migliario et al.^57^	930	1.57	$1580\;{\mathrm{mW}/\mathrm{cm}}^{2}$ 1 W,1 s, $0.63\;{\mathrm{cm}}^{2}$	Preosteoblast
Migliario et al.^57^	930	78.7	$1580\;{\mathrm{mW}/\mathrm{cm}}^{2}$ 1 W, 50 s, $0.63\;{\mathrm{cm}}^{2}$	Preosteoblast
Skopin et al.^58^	980	5.9	$49\;{\mathrm{mW}/\mathrm{cm}}^{2}$	Fibroblast
Skopin et al.^58^	980	14.4	$120\;{\mathrm{mW}/\mathrm{cm}}^{2}$	Fibroblast
Zhang et al.^53^	628	9.0	$11.4\;{\mathrm{mW}/\mathrm{cm}}^{2}$ 15 mW, distance of 0.75 cm	Fibroblast
Khadra et al.^75^	830	0.75	$8.4\;{\mathrm{mW}/\mathrm{cm}}^{2}$ 84 mW, $10\;{\mathrm{cm}}^{2}$, 360 s, 9 cm distance to cells	Fibroblast
Wang et al.^73^	980	20	$16\;{\mathrm{mW}/\mathrm{cm}}^{2}$ $4\;{\mathrm{cm}}^{2}$, 1 W	Adipose stem cells

**Table 9:** Ineffective PBM treatment *in vivo* on tissues with higher number of mitochondria.

Authors	Wavelength (nm)	Fluence ($J/{\mathrm{cm}}^{2}$)	Irradiance	Tissue type
Oron et al.^62^^,^^63^	810	0.3	$2.5\;{\mathrm{mW}/\mathrm{cm}}^{2}$ 5 mW, area of irradiation of $1.1\;{\mathrm{cm}}^{2}$	Heart
Oron et al.^62^^,^^63^	810	0.3	$25\;{\mathrm{mW}/\mathrm{cm}}^{2}$ 5 mW, area of irradiation of $1.1\;{\mathrm{cm}}^{2}$	Heart
Salehpour et al.^59^	660	4	$4.75\;{W/\mathrm{cm}}^{2}$ 10 Hz, $4.75\;{W/\mathrm{cm}}^{2}$, 88% duty cycle, 200 mW	Brain
Salehpour et al.^59^	810	4	$4.75\;{W/\mathrm{cm}}^{2}$ 10 Hz, $4.75\;{W/\mathrm{cm}}^{2}$, 88% duty cycle, 200 mW	Brain
Wu et al.^78^	980	36	$15\;{\mathrm{mW}/\mathrm{cm}}^{2}$	Brain
Alves et al.^25^	808	142.4	$3.57\;{W/\mathrm{cm}}^{2}$ 4 J, 50 mW, $0.028\;{\mathrm{cm}}^{2}$, 80 s per point	Arthritis
Lopes-Martins et al.^18^	655	2.5	$31.25\;{\mathrm{mW}/\mathrm{cm}}^{2}$ 2.5 mW, spot area $0.08\;{\mathrm{cm}}^{2}$, 25 mW, 160 s, 2.5 mW	Muscle

**Table 10:** Ineffective PBM treatment *in vivo* on tissues with lower number of mitochondria.

Authors	Wavelength (nm)	Fluence ($J/{\mathrm{cm}}^{2}$)	Irradiance	Tissue type
Lanzafame et al.^15^	670	5.0	$0.7\;{\mathrm{mW}/\mathrm{cm}}^{2}$	Ulcers (wound healing)
Lanzafame et al.^15^	670	5.0	$2.0\;{\mathrm{mW}/\mathrm{cm}}^{2}$	Ulcers (wound healing)
Gal et al.^82^	670	5.0	$15\;{\mathrm{mW}/\mathrm{cm}}^{2}$	Wound healing
Lanzafame et al.^15^	670	5.0	$40\;{\mathrm{mW}/\mathrm{cm}}^{2}$	Wound healing
Prabhu et al.^81^	632	10	$4.0\;{\mathrm{mW}/\mathrm{cm}}^{2}$ 7 mw, $1.75\;{\mathrm{cm}}^{2}$	Wound healing
Al-Watban et al.^83^	670	9.0	$130\;{\mathrm{mW}/\mathrm{cm}}^{2}$ 200 mW, $1.534\;{\mathrm{cm}}^{2}$	Wound healing
Al-Watban et al.^83^	670	19	$130\;{\mathrm{mW}/\mathrm{cm}}^{2}$ 200 mW, $1.534\;{\mathrm{cm}}^{2}$	Wound healing
Kilik et al.^88^	636	5	$1\;{\mathrm{mW}/\mathrm{cm}}^{2}$ Probe to wound 10 cm	Wound healing

### 4.1 Wavelength

Wavelength affects tissue penetration. Shorter wavelengths (600 to 700 nm) are considered best to treat superficial tissue, whereas longer wavelengths (780 to 950 nm) are preferred to treat deeper tissues. Red wavelengths penetrate 0.5 to 1 mm and near-infrared energy penetrates 2 mm before losing 37% of its intensity.^89^^–^^91^

The infrared wavelengths show better effects on bone repair compared to red wavelengths because red light has less capacity to penetrate compared to the infrared laser.

According to Karu,^8^ wavelengths between 700 and 770 nm do not have any significant activity. Wu et al.^78^ used a 730-nm laser on TBI in mice and found it to be ineffective while 660 and 810 nm lasers were effective. Gupta et al.^92^ carried out a similar comparison on wound healing in mice and again found that 660- and 810-nm lasers were effective, while a 730-nm laser was not effective.

Barbosa et al.^20^ concluded that the PBM effects of NIR were effective for more than 14 days, whereas the effects of red wavelength are lost after 14 days.

The combination of two wavelengths gives an additional effect of PBM. When comparing 830 and 685 nm, Mendez et al.^21^ found that 830 nm offered better results. Much work still remains to define the optimal wavelengths. Nevertheless, NIR wavelengths are preferable for deep tissues and targets within the body, which require substantial doses of light.

### 4.2 Laser Versus Noncoherent Light

Both coherent lasers and noncoherent LEDs are used in PBMT. Laser beams are collimated and the light is more likely to be forward scattered within the tissue than noncollimated LED light.^5^ This means that the penetration depth is likely to be deeper with lasers provided all the other characteristics are identical. Moreover, lasers emit coherent light, while LED light is noncoherent. The coherence length is higher for smaller bandwidths. For instance, gas lasers such as He–Ne laser have very long coherence lengths. Diode lasers have somewhat greater bandwidths and consequently shorter coherence lengths. When coherent laser light interacts with tissue, small imperfections in the tissue structure lead to different phases occurring in the individual wavefronts leading to mutual interference patterns. These interference patterns are called “laser speckles” and the size of the speckles is related to the light wavelength. In the visible range, the sizes are less than 1  μm. Subcellular organelles (such as mitochondria) have dimensions of this order and a theory proposes that the laser speckles are better to stimulate mitochondria than noncoherent LED light.^93^^–^^95^ A recent review concluded that there were no substantial differences between lasers and LEDs for PBM applications provided all the other light parameters were equal.^96^

### 4.3 Fluence and Irradiance

The photon intensity i.e., irradiance ($W/{\mathrm{cm}}^{2}$ or spectral irradiance), must be adequate. Using higher intensity, the photon energy will be transformed to excessive heat in the target tissue and, using lower intensity, photons absorption will be insufficient to achieve the goal.

The dose also must be adequate ($J/{\mathrm{cm}}^{2}$). Using low irradiance and prolonging the irradiation time to achieve the ideal fluence or dose will not give an adequate final result. The Bunsen–Roscoe law of reciprocity, termed the second law of photobiology,^97^ does not hold true for low incident power densities.

There is no fixed value of dose or fluence that always produces a positive PBM effect. Even within different studies on the same animal models, there can be contradictory findings. For instance, three papers looked at peri-implant bone regeneration after PBM. Menezes et al.^98^ found that $20\;{J/\mathrm{cm}}^{2}$ was the best dose to deliver, whereas Massotti et al.^22^ and Mayer et al.^23^ found that $20\;{J/\mathrm{cm}}^{2}$ was the worst dose to deliver.

The optimal doses are directly related to different factors:

• Wavelength
• Type of treatment being delivered: pain relief, wound healing, or tissue regeneration
• Power density or irradiance
• Energy density or fluence
• Depth of the target tissue being treated
• Spot size of the beam reaching the tissue surface and the actual target tissue.

**Fig. 1:**
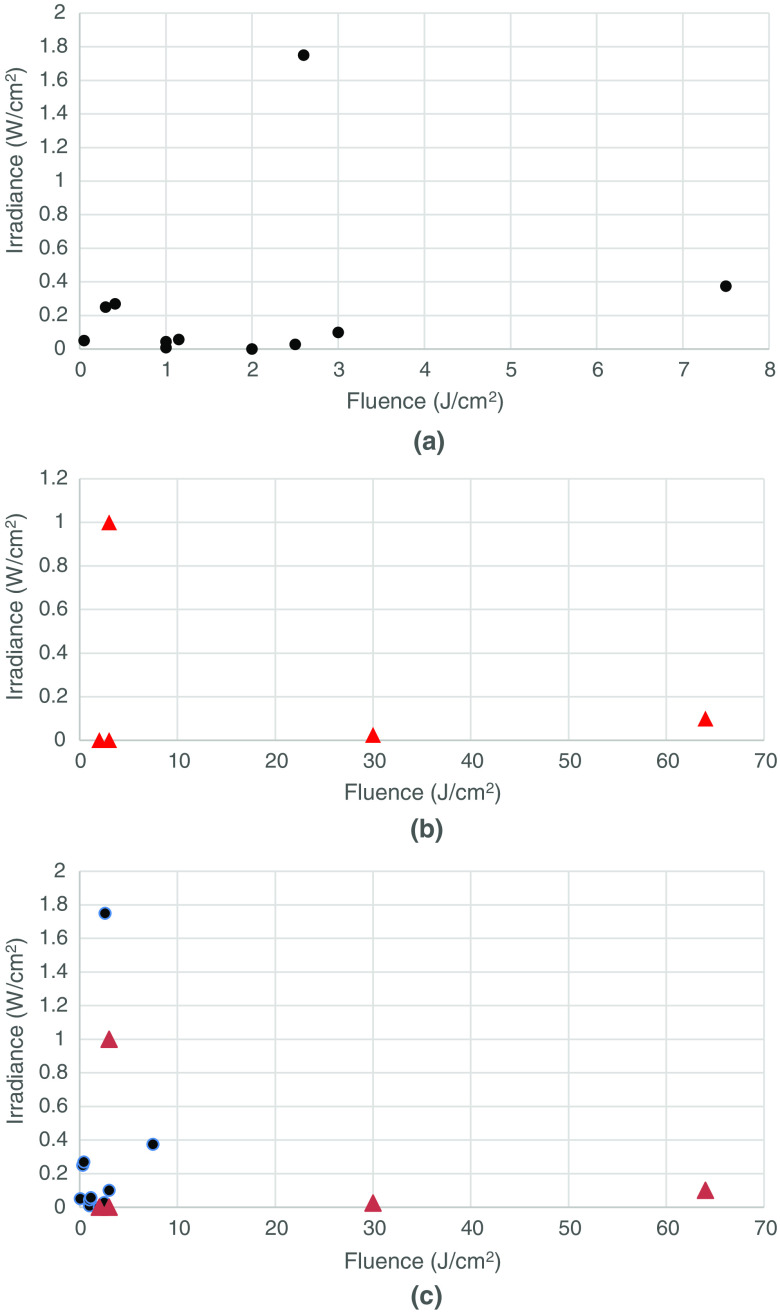
Studies on PBM of cells *in vitro* with higher numbers of mitochondria. (a) Effective (positive studies), (b) ineffective (negative studies), and (c) combination of effective (positive studies), and ineffective (negative studies).

**Fig. 2:**
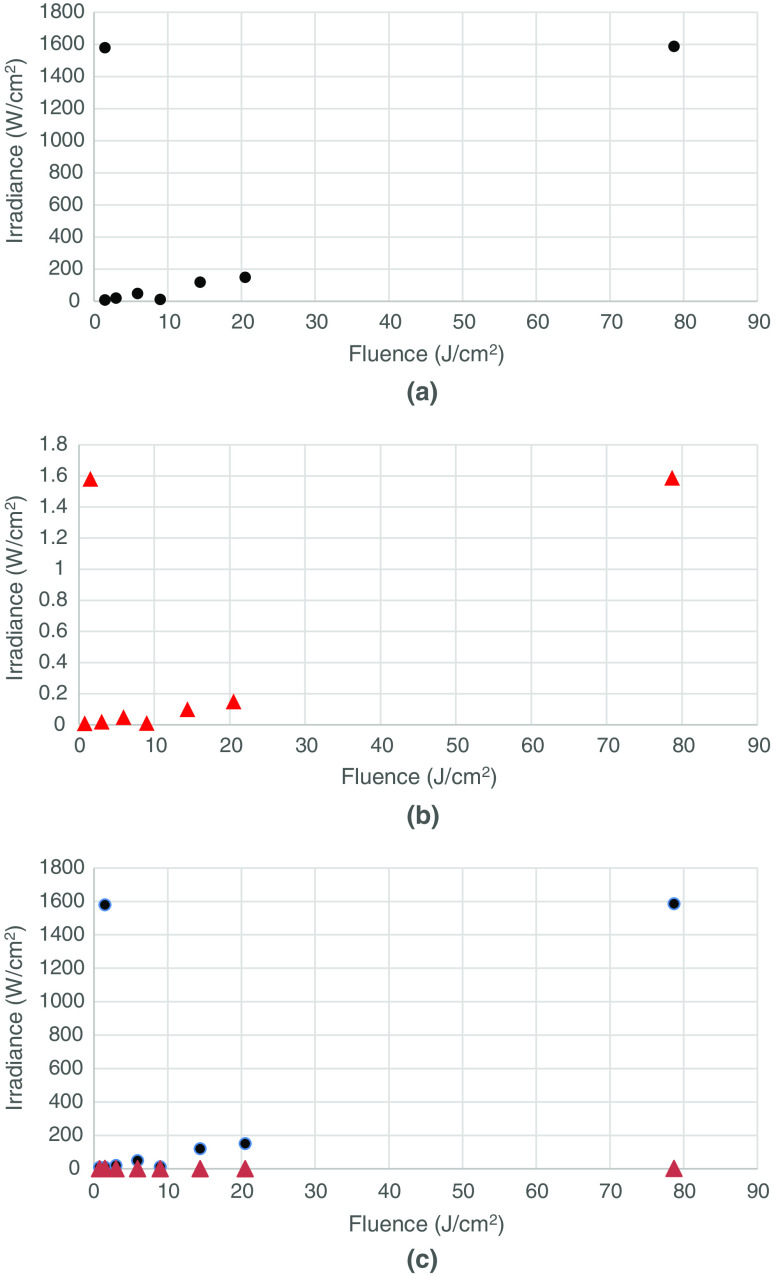
Studies on PBM of cells *in vitro* with lower numbers of mitochondria. (a) Effective (positive studies), (b) ineffective (negative studies), and (c) combination of effective (positive studies) and ineffective (negative studies).

**Fig. 3:**
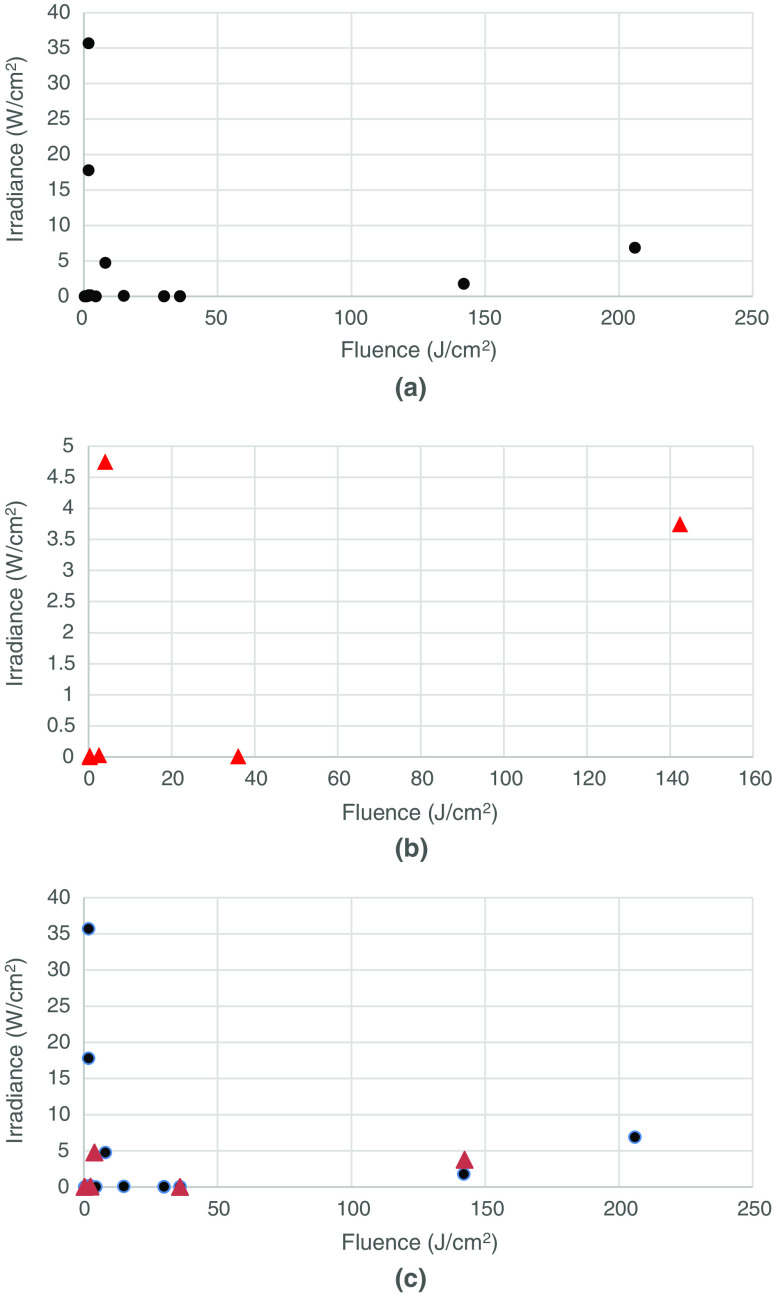
Studies on PBM of tissues *in vivo* with higher numbers of mitochondria. (a) Effective (positive studies), (b) ineffective (negative studies), and (c) combination of effective (positive studies) and ineffective (negative studies).

**Fig. 4:**
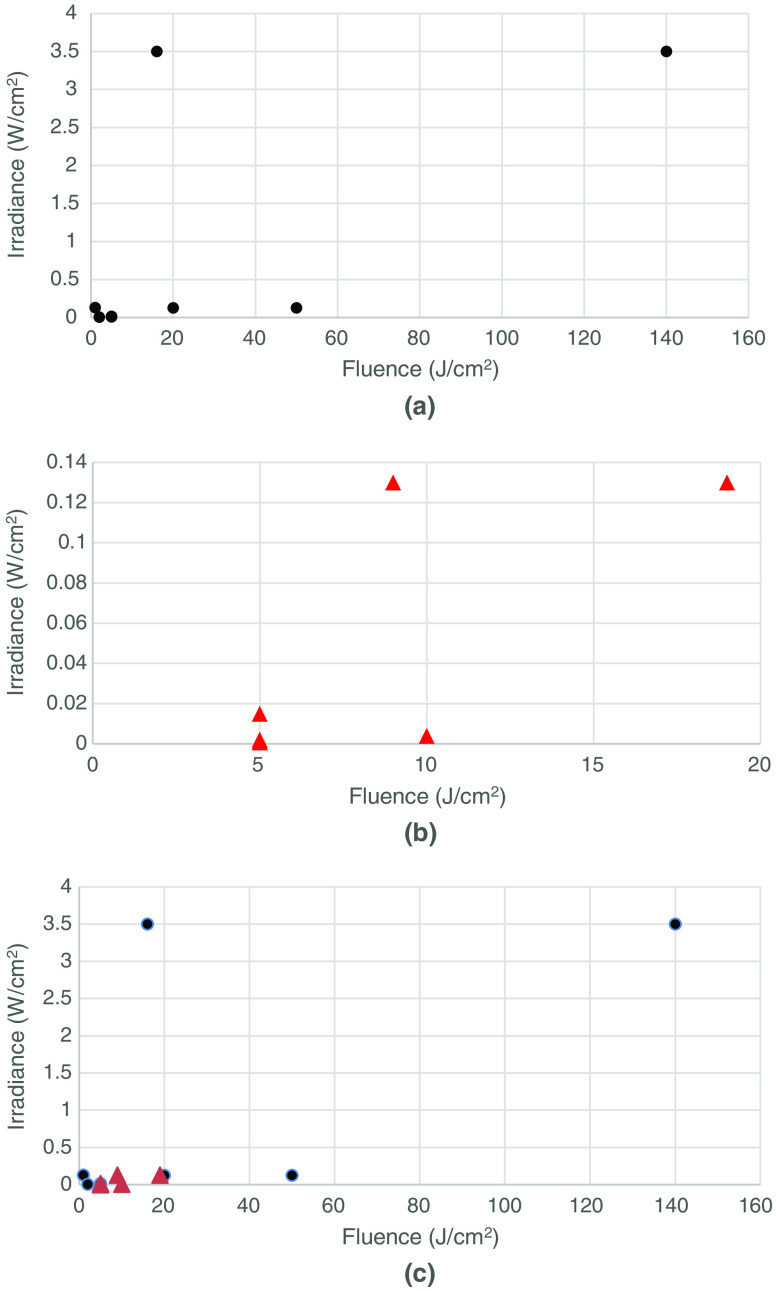
Studies on PBM of tissues *in vivo* with lower numbers of mitochondria. (a) Effective (positive studies), (b) ineffective (negative studies), and (c) combination of effective (positive studies) and ineffective (negative studies).

In an attempt to determine whether the delivered fluence ($J/{\mathrm{cm}}^{2}$) was more or less important than the irradiance ($\mathrm{mW}/{\mathrm{cm}}^{2}$), we constructed scatter plots (Figs. 1–4) of both the effective and ineffective studies arranged according to our categorization of the studies in Tables 2–9.

#### 4.3.1 In vitro studies

Figure 1(a) shows the plot of *in vitro* studies in cells with higher numbers of mitochondria, whereas Fig. 1(b) shows the corresponding plot for cells with lower numbers of mitochondria. The following observations can be made. In all the effective studies, the fluence was relatively low ($<7.5\;{J/\mathrm{cm}}^{2}$) and in several cases, less than $1\;{J/\mathrm{cm}}^{2}$. However, in the ineffective studies, the fluence values were larger (all $>3\;{J/\mathrm{cm}}^{2}$), and in two cases, very large values (30 and $65\;{J/\mathrm{cm}}^{2}$). There were more studies in the effective group (11) than in the ineffective group (5). This suggests that high-mitochondrial cells respond well to PBM and that ineffective studies are more likely to be due to over-dosing than to under-dosing.

Figure 2(a) shows the effective *in vitro* studies in cells with lower mitochondrial numbers. Again, the positive studies outweigh the negative studies [Fig. 2(b)] (15 to 8). The fluence values in the positive studies in the lower mitochondrial number subgroup appeared to be overall higher than the fluences used in the positive studies in the higher mitochondrial number subgroup. The fluences used in the negative studies in the lower mitochondrial number subgroup were only a little higher than those in the positive studies, suggesting that over-dosing was not such a big problem as it was in the higher mitochondrial number subgroup [Fig. 1(b)]. There were three positive studies that used relatively high irradiances ($>1.5\;{W/\mathrm{cm}}^{2}$), as opposed to only one study in the positive high-mitochondrial subgroup.

#### 4.3.2 In vivo studies

Figure 3(a) shows the plot of effective or positive studies *in vivo* on tissues composed of cells with higher numbers of mitochondria, whereas Fig. 3(b) shows the corresponding plot for ineffective or negative studies on tissues composed of higher mitochondrial number cells. Here, a difference is seen when comparing the two plots and with the analogous two plots from the *in vitro* studies. In the *in vivo* case, the fluence values in the effective studies subgroup [Fig. 3(a)] are higher than those in the ineffective studies subgroup [Fig. 3(b)]. This is the opposite of what was found in the *in vitro* case with cultured cells [compare Figs. 1(a) with 1(b)]. Hence, these observations tend to suggest that failure, *in vivo*, could be due to under-dosing while failure, *in vitro*, could equally well be due to over-dosing. *In vivo*, the depth of the tissue is important, while cells, *in vitro* culture, are generally a single monolayer. It is a fact that tissues with higher numbers of mitochondria (brain, heart, muscles, inflammatory cells) tend to be deeper within the body than tissues with lower numbers of mitochondria (skin, tendons, cartilage). There are, of course, some exceptions (bones and bone marrow), which have lower numbers of mitochondria but are still deep within the body.

Figure 4(a) shows the plot of effective treatment in tissue with a lower number of mitochondria, whereas Fig. 4(b) shows the plot of ineffective treatment on tissue with a lower number of mitochondria.

The following observation can be made:

The fluence values used in the positive studies are much higher than those in the negative studies, particularly when the tissue is deeper (such as bone). In addition, some studies used very low fluences of less than $1\;{J/\mathrm{cm}}^{2}$ to treat superficial tissue (wound healing) and had positive results.

Fluences used in the negative studies are generally less than $10\;{J/\mathrm{cm}}^{2}$, most of them used low irradiance. There are three studies that use lower fluence in combination with higher irradiance and produced positive results.

This would suggest that ineffective studies for tissue with lower mitochondria are more likely to be due to under-dosing rather than over-dosing. Fluence and irradiance are both important in determining the success of *in vivo* studies.

## 5 Conclusions

The limitation of this analysis was the relatively small number of studies that passed our inclusion and exclusion criteria. Nevertheless, some tentative conclusions can be drawn from the analysis that we can at least propose for other researchers to confirm or refute, as more well-documented studies continue to be published in the coming years.

1. Cells with higher numbers of mitochondria respond better to PBM than cells with lower numbers of mitochondria.
2. Ineffective studies on cells with higher numbers of mitochondria are as likely to be due to over-dosing as they are to under-dosing.
3. It is less likely that ineffective studies in cells with lower numbers of mitochondria will be due to over-dosing.
4. The fluence delivered is more important in determining the success or failure of an *in vitro* study than the irradiance employed.
5. Tissues with higher numbers of mitochondria tend to be deeper within the body than tissues with lower numbers of mitochondria, therefore, over-dosing is less likely.
6. Ineffective studies *in vivo* are more likely to be due to under-dosing regardless of the number of mitochondria.
